# Improving upon the ‘July effect’: a collaborative, interdisciplinary orientation for internal medicine interns

**DOI:** 10.3402/meo.v18i0.23249

**Published:** 2013-12-23

**Authors:** Mary Wright, Celina G. Mankey, Beth W. Miller

**Affiliations:** 1 University Medical Center Brackenridge, Seton Healthcare Family, Austin, TX, USA; 2 Internal Medicine Program, University of Texas Southwestern Austin, Austin, TX, USA

US teaching hospitals orient interns each year, usually in the summer months (June–July). These new physicians have usually just finished with their medical school training and do not have any other clinical experience outside of medical school (i.e., without supervision). They are asked to begin seeing patients on day 1 of their internship without having formed relationships with other clinical staff such as nursing and without knowing the hospital system. It is also commonly believed that the ‘quality of health care decreases during trainee academic year-end changeovers’ (1). Yet the overwhelming phenomenon continues.

Traditionally intern orientation has consisted of long hours sitting through lectures resulting in information overload, followed by a brief tour of the hospital with their attending physician or chief resident. The interns begin their clinical experiences with patient responsibilities without any collegial relationships with the clinical team who carry out their orders, nor any familiarity with the hospital protocols or culture and flow of hospital life. Many interns admit later that the first few months were an overwhelming experience and one that could certainly fail to meet patient needs.

Experienced nurses in teaching hospitals are also challenged during this time. As part of this phenomenon, nurses often become the middle-person advocating for the patient and sometimes the new intern. Interns may also be hesitant to question the suggestions of nurses and are unfamiliar with nursing roles and responsibilities. Certainly during this critical time, there is no opportunity to leverage team building.

Building inter-professional collaboration between physicians and nurses is just what is needed in the very beginning of July to exponentially improve upon this introductory transition within and between professional cultures while still ‘containing cost and insuring better patient outcomes’ (2). Literature suggests an active orientation, which provides improved communication between caregivers, and growth of collegial relationships between physicians and nurses will ultimately improve patient outcomes (3). High-quality communication and strong relationships are the foundation for coordinated action in high-stress hospital settings (3).

From 2010 to 2012, the University of Texas Southwestern Austin internal medicine program and nursing at University Medical Center Brackenridge developed an intern orientation program that was multi-disciplinary and collaborative in nature. Our hypothesis was that the program would engage the interns and foster the development of collegial relationships with nursing and support staff as well as provide real-time clinical practice and feedback through simulation. We also believed that interns would feel more adjusted to the program and hospital system.

## Methods

Faced with consistent concerns that arise every July, a survey was formulated and distributed to the 2009 interns, approximately 8 months into their first year of internship to query ‘what they wish they had known the first week of their clinical rotation at their hospital facility’. The interns’ (*n*=20) concerns echoed what the nurses had noted. Responses included simple concerns such as where the bathrooms were located, to the complex early signs of sepsis for the neutropenic patient. Based on findings from the survey, we launched a new collaborative interdisciplinary orientation program in 2010 to assimilate interns into the hospital culture. Each year improvements have been made to the orientation program.

In 2010 Nursing at University medical Center Brackenridge (UMCB) worked with internal medicine leadership to improve the intern orientation program. During the first year the nurses adopted an intern buddy, gave them a pocket sized welcome resource booklet (Table 1), and toured the interns providing special unit information, from the simple ‘flow’ of the unit to the complexities of the patient population. Expounding on the research that simulation is a safe realistic way to capture ‘real life’ events and an appropriate way to teach new professionals (3), several nurses assisted in a 4-hour ‘hands-on training’ in the simulation center.

**Table 1:** Welcome booklet for internal medicine interns

The laminated booklet has been provided to be a convenient resource guide to alleviate the anxiety of working in a new hospital. It fits nicely in the intern's white coat pocket. Booklet contents include:
Phone numbers to each Nursing Unit The direct number for each Charge Nurse Critical Response Team number Radiology phone numbers Interpreter phone number Phone numbers to all residents, faculty, attendings, and consults Phone number to infection control – for Isolation consults Door codes – for bathrooms and copy machines Guide to Caregivers Scrub Colors – to easily identify a nurse, respiratory therapist, physical therapist, pharmacy, clerks, housekeeping nursing assistants, and radiology List of emergency Codes such as Code Red, Code Twist, and Code Orange Fire Response – RACEE Brief scope of service for each acute care unit to aid in proper patient placement. Sepsis: Early Recognition Algorithm Directions for listening to Radiology Reports and looking at actual films Directions for Transcription Do Not Use abbreviation list High Reliability Healing/Core Measures

In 2011, the orientation program grew with more collaborative efforts including the addition of ancillary departments and the inclusion of more simulation cases with nurse buddies during the orientation and collaborative simulations throughout the year. Computer order entry education was also added with nurses acting as proctors.

We continue to expand upon the program every year due to positive feedback. In 2012, the nurse–buddy program was extended. The interns were matched with a ‘buddy–nurse’ with similar personal interests and with a nurse who would be on a unit/time that they would frequent during the first month. Further simulation experiences were also added.

Orientation week included a nursing unit tour, ancillary fair, and three afternoons in simulation and computer labs with nurse–buddies. Attending physicians sponsored a luncheon, which featured a collaborative doctor/nurse bingo. Following lunch, the afternoons consisted of collaborative simulation sessions that included a procedures lab, an atrial fibrillation case, a women's health workshop, a breaking bad news case, an agitated and/or angry patient case, and a case on doing a correct hand-off and calling in a consult. The ancillary fair introduced the intern to other members of the health care team and informed them of the hospital's goals, initiatives, and culture. The intern visited booths with groups such as the safety team discussing high reliability training and patient representatives discussing the language of caring. The intern met case managers, social workers, rehabilitation services, the infection control nurse, and others in small groups with their nurse–buddy who provided information about their department and how they could help the intern provide the best care for their patients.

This program received exemption status from the hospital's institutional review board.

## Results

The initial collaborative orientation in 2010 set the tone for more positive relationships throughout the year. This first attempt received positive results from interns and nurses, and requests from ancillary personnel to be included next year. Findings (case reports), similar to Ushiro's criteria (4), showed improved communication between nursing and interns such as better collegiality when nurses and physicians greeted each other daily, showing concern for each other when they were tired, and freely exchanging patient information.

All of the interns completed an anonymous, online survey at the end of the orientation program in 2011 (*n*=21). It was rated very highly (100% rated it 4–5 on a scale of 1–5), and the interns reported that they found the orientation very/extremely helpful to interact with their nursing colleagues during orientation. They were also interested in spending more time in the simulation center. They rated the ancillary fair very highly as well. There have been case reports of interns connecting with their nurse buddies to discuss issues on the wards. Three month evaluations from interns, nurses, and faculty suggest that this program was extremely helpful in orienting the interns to the program and hospital, in helping them develop better relationships with nursing and ancillary staff, and possibly helping them feel less anxious about starting an internship.

The nurses were also confident that the more interactive and collaborative approach would be an improvement and have been overwhelmed by the improved interdisciplinary trust and relationships built by this approach. According to post orientation surveys, nurses felt collegial relationships were formed, which increased nurse comfort giving the interns recommendations. Nurses felt the interns understood nurse workflow, and nurses learned valuable information doing simulations with the interns. In addition, unit nurses have reported that interns are more confident and are working more collaboratively with nurses and other healthcare professionals. Nurses made a commitment to check in with their intern buddies, complete quarterly evaluations, and to assist in on-going simulations throughout the year.

## Conclusion

Intern orientation without nursing involvement fails to leverage team building at a critical time. Literature suggests that an interdisciplinary and active orientation, which can lead to improved communication between caregivers and growth of collegial relationships between physicians and nurses, will ultimately improve patient outcomes.

We plan to expand our intern orientation program to other residency programs for the upcoming year, and continue to further develop our program. Given the positive feedback that we have received from all participants (nurses, interns, ancillary staff, faculty), we hope to continue to share our innovative program with others around the country so that other programs can hopefully develop and implement similar inter-disciplinary intern orientations in their hospitals.

In the end, everyone, especially patients, benefit from improved collegiality and relationships that are developed through this type of program. We also believe that patient safety and quality of care could improve with these inter-disciplinary programs. Further studies would be needed to determine if these programs improve patient outcomes.

### Financial considerations

This program received the support of the Chief Operating Officer, Chief Medical Officer, and the Chief Nursing Officer at University Medical Center Brackenridge who encouraged department directors to allow staff to participate. Many who participated were salaried employees who arranged their schedules to participate. Staff nurses used education delivery hours that were included in the yearly budget. On-going simulations hours were transferred to the IM program budget. IM faculty used educational value units (EVUs) that were also included in the yearly budget for their time during the simulation cases.
